# Supplementary material for: Highly Specific and Rapid Detection of Hepatitis C Virus Using RT-LAMP-Coupled CRISPR–Cas12 Assay
Source: Diagnostics (Basel). 2022 Jun 23;12(7):1524. doi: 10.3390/diagnostics12071524 (PMC9317538; doi:10.3390/diagnostics12071524)
Supplement: Supplementary file 1 [file diagnostics-12-01524-s001.zip › diagnostics-1767661-supplementary.pdf]

diagnostics

MDPI

Supplementary Materials

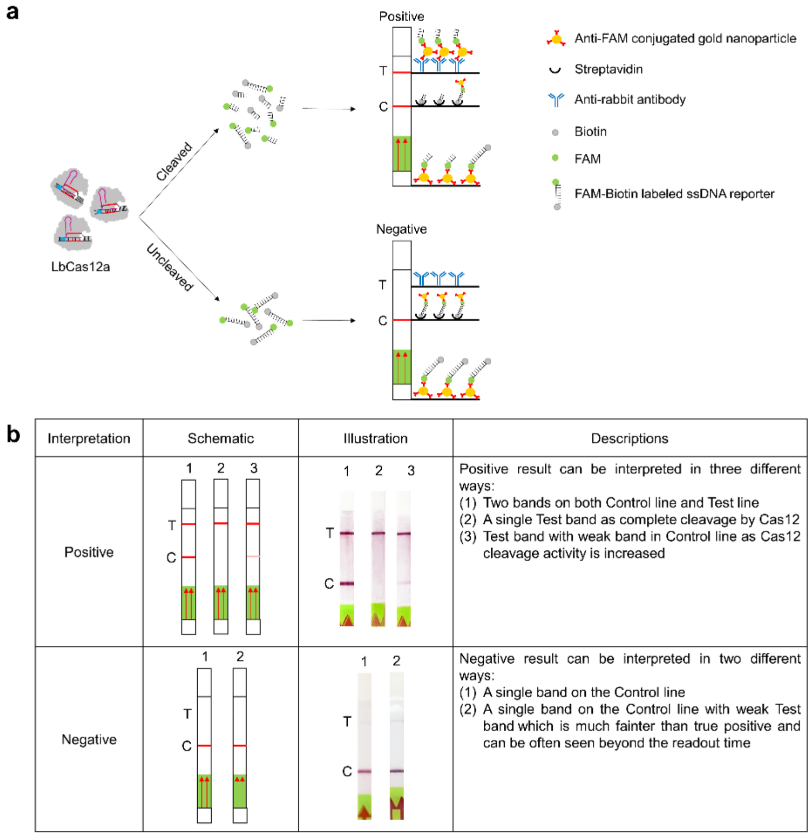

Figure S1. Interpretation of results for CRISPR-Cas12 assay with lateral flow-based readout. (a) Il-lustration of the principle of lateral flow assay. After recognition of the target sequence by Cas12, the ssDNA reporters labeling with carboxyfluorescein (FAM) and biotin were cleaved. Then, the cleaved-FAM binds to anti-FAM conjugated with a gold nanoparticle on the sample pad. After flow-ing through the strip, the cleaved-biotin is captured by streptavidin on the Control line and anti-FAM is captured by anti-rabbit antibody present on the Test line. This can be interpreted as a posi-tive result. In a negative result, there is no cleavage of the reporter by Cas12. Therefore, biotin on an intact reporter is captured by streptavidin on the Control line; (b) Result interpretation of the RT-LAMP-coupled CRISPR-Cas12 assay combining with a lateral flow-based readout. T, Test line; C, Control line.

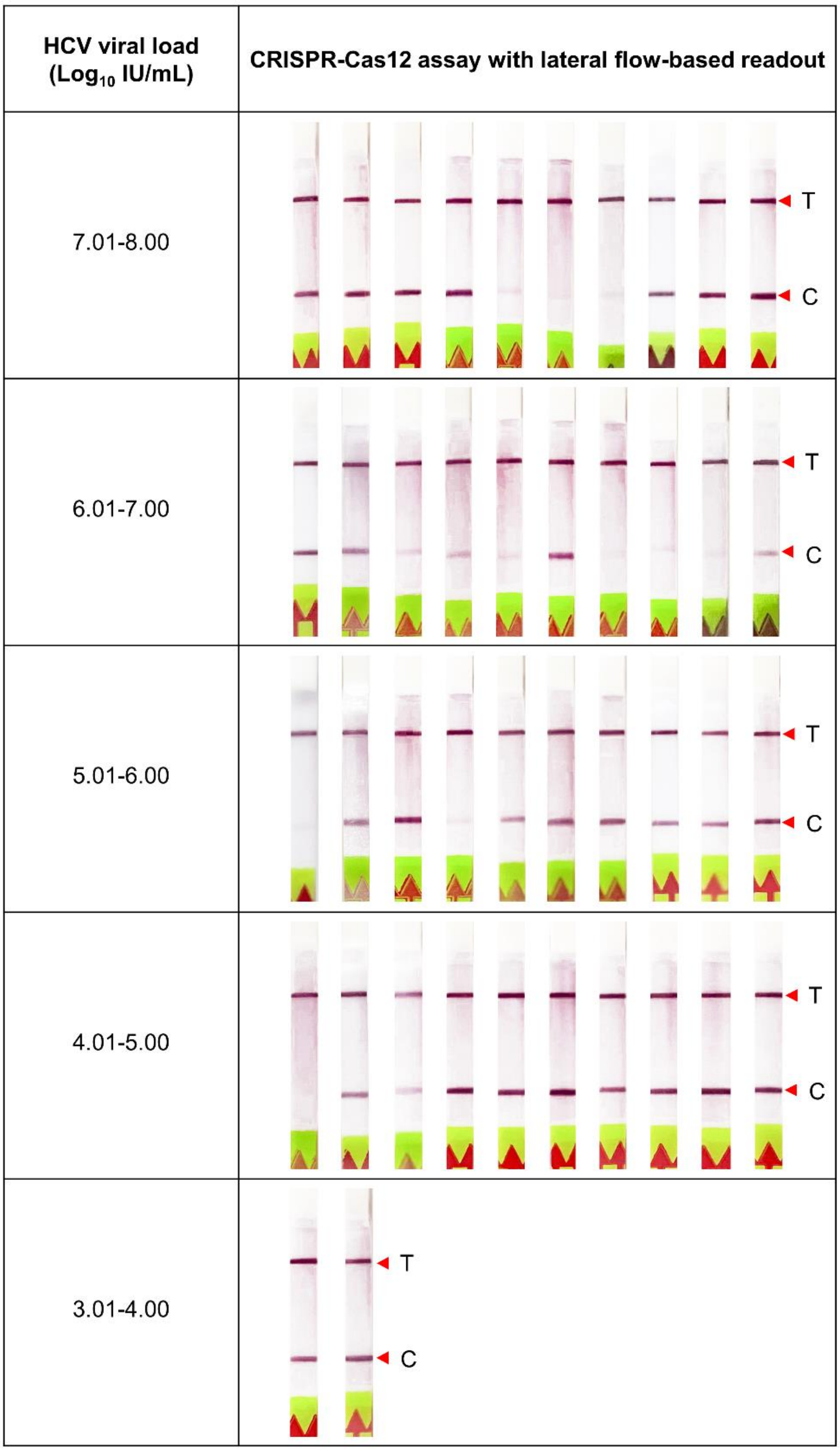

Figure S2. Evaluation of CRISPR-Cas12 assay with lateral flow-based readout according to HCV viral loads. T, Test line; C, Control line.
